# Inhibition of mTOR-Dependent Autophagy Sensitizes Leukemic Cells to Cytarabine-Induced Apoptotic Death

**DOI:** 10.1371/journal.pone.0094374

**Published:** 2014-04-08

**Authors:** Mihajlo Bosnjak, Biljana Ristic, Katarina Arsikin, Aleksandar Mircic, Violeta Suzin-Zivkovic, Vladimir Perovic, Andrija Bogdanovic, Verica Paunovic, Ivanka Markovic, Vladimir Bumbasirevic, Vladimir Trajkovic, Ljubica Harhaji-Trajkovic

**Affiliations:** 1 Institute of Histology and Embryology, School of Medicine, University of Belgrade, Belgrade, Serbia; 2 Institute of Microbiology and Immunology, School of Medicine, University of Belgrade, Dr. Subotica 1, Belgrade, Serbia; 3 Clinic of Hematology, Clinical Center of Serbia, School of Medicine, University of Belgrade, Belgrade, Serbia; 4 Institute of Medical and Clinical Biochemistry, School of Medicine, University of Belgrade, Belgrade, Serbia; 5 Institute for Biological Research, University of Belgrade, Belgrade, Serbia; Complutense University, Spain

## Abstract

The present study investigated the role of autophagy, a cellular self-digestion process, in the cytotoxicity of antileukemic drug cytarabine towards human leukemic cell lines (REH, HL-60, MOLT-4) and peripheral blood mononuclear cells from leukemic patients. The induction of autophagy was confirmed by acridine orange staining of intracellular acidic vesicles, electron microscopy visualization of autophagic vacuoles, as well as by the increase in autophagic proteolysis and autophagic flux, demonstrated by immunoblot analysis of p62 downregulation and LC3-I conversion to autophagosome-associated LC3-II in the presence of proteolysis inhibitors, respectively. Moreover, the expression of autophagy-related genes Atg4, Atg5 and Atg7 was stimulated by cytarabine in REH cells. Cytarabine reduced the phosphorylation of the major negative regulator of autophagy, mammalian target of rapamycin (mTOR), and its downstream target p70S6 kinase in REH cells, which was associated with downregulation of mTOR activator Akt and activation of extracellular signal- regulated kinase. Cytarabine had no effect on the activation of mTOR inhibitor AMP-activated protein kinase. Leucine, an mTOR activator, reduced both cytarabine-induced autophagy and cytotoxicity. Accordingly, pharmacological downregulation of autophagy with bafilomycin A1 and chloroquine, or RNA interference-mediated knockdown of LC3β or p62, markedly increased oxidative stress, mitochondrial depolarization, caspase activation and subsequent DNA fragmentation and apoptotic death in cytarabine-treated REH cells. Cytarabine also induced mTOR-dependent cytoprotective autophagy in HL-60 and MOLT-4 leukemic cell lines, as well as primary leukemic cells, but not normal leukocytes. These data suggest that the therapeutic efficiency of cytarabine in leukemic patients could be increased by the inhibition of the mTOR-dependent autophagic response.

## Introduction

Cytarabine (cytosine arabinoside, arabinofuranosyl cytidine) is a chemotherapeutic drug used alone or in combination with other antineoplastic agents to treat different forms of leukemia. As an analog of deoxycytidine, this antimetabolite drug incorporates into human DNA and consequently kills leukemic cells by interfering with DNA and RNA synthesis [1]. Low permeability of cytarabine across the cell membrane, need for biological activation through phosphorylation and rapid deamination into inactive 1-β-d-arabinofuranosyluracil require high cytarabine doses in order to achieve satisfactory antileukemic effect [2]. However, treatment with high doses of the drug has been associated with severe side effects including cerebellar toxicity, leukopenia, thrombocytopenia, anemia, gastrointestinal disturbances and fatal toxicities [3]. To prevent the adverse effects and improve sensitivity of leukemia cells, cytarabine has been combined with different agents capable of modulating its stability, lipophilicity or apoptotic response of cancer cells [2].

The induction of macroautophagy (referred to hereafter as autophagy), a catabolic process for degradation and recycling of the cell's own unnecessary or dysfunctional components [4], has recently been implicated in regulation of leukemic cell death triggered by anticancer drugs [5]–[15]. Autophagy involves sequestration of intracellular content in double-membraned autophagosomes, followed by their fusion with lysosomes and formation of single-membraned autophagolysosomes, in which the internal content is degraded by acidic lysosomal hydrolases [4]. Autophagy depends on the hierarchically ordered activity of autophagy-related (Atg) proteins, controlled by the main autophagy repressor, mammalian target of rapamycin (mTOR) [4]. This serine/threonine kinase is activated by phosphoinositide 3-kinase (PI3K)/Akt pathway and inhibited by the intracellular energy sensor AMP-activated protein kinase (AMPK) [4]. In addition, the activation of extracellular signal-regulated kinase (ERK) has been described as a non-canonical signal downstream of AMPK that contributes to mTOR-dependent induction of autophagy in certain conditions [16]. Under stress conditions such as nutrient deprivation, hypoxia, oxidative stress and DNA damage, autophagy could provide energy for maintaining essential cellular metabolism and/or directly interfere with apoptotic/necrotic cell death pathways [17]. However, in certain conditions, autophagy can also stimulate apoptosis [18] or function as an alternative cell-death pathway (programmed cell death type II) [19]. Accordingly, autophagy can either promote cell death or serve as a survival mechanism in leukemic cells treated with different anticancer drugs [20], [21]. It has recently been reported that tyrosine kinase inhibitor imatinib mesylate and high-mobility group protein B1 promote resistance of cytarabine-treated leukemic cells through induction of autophagy [22], [23]. However, to the best of our knowledge, no study so far has investigated the ability of cytarabine to induce autophagic response in cancer cells, or the possible role of cytarabine-triggered autophagy in regulation of cancer cell death.

In the present study, we demonstrate that cytarabine induces autophagy in leukemic cell lines and primary leukemic cells, but not healthy leukocytes, by inhibiting the main negative autophagy regulator, mammalian target of rapamycin (mTOR) [4]. Moreover, pharmacological and genetic inhibition of autophagy sensitized leukemic cells to cytarabine-induced apoptosis.

## Materials and Methods

### Cell culture

Human lymphoblastic leukemia cell line REH (ATCC CRL-8286) was obtained from American Type Culture Collection (Manassas, VA), while human promyelocytic leukemia cell line HL-60 (ECACC 98070106) was purchased from the European Collection of Animal Cell Cultures (Salisbury, UK). Peripheral blood mononuclear cells (PBMC) were obtained from venous blood of three patients with blastic transformation as the first presentation of the chronic myeloid leukemia (CML), with more than 10^8^/l WBC count and myeloblast/promyelocyte count > 50%. The diagnosis was established at the Outpatient Clinic of the Outpatient & Diagnostic Department, Clinic of Hematology, Clinical Centre of Serbia (Serbia, Belgrade), according to the diagnostic criteria for classification of tumors of hematopoietic and lymphoid tissue [24]. Control PBMC were obtained from three healthy volunteers, age- and sex-matched with leukemic patients. The study was conducted in accordance with the Declaration of Helsinki and approved by the Ethical Committee of the Clinical Centre of Serbia and the Ethical Committee of the School of Medicine, University of Belgrade. Each volunteer provided a written consent for participation in the study after being informed about all the details of the study. All patients provided two informed written consents, one general concerning diagnostic procedures, and another one concerning the scientific analysis, because the samples were taken during regular diagnostic workup. Blood draws were conducted with syringes containing 10% (v/v) of 3.8% sodium citrate as an anticoagulant. PBMC were isolated by density gradient centrifugation using LymphoPrep (Axis Shield, Oslo, Norway) and immediately used for experiments. The cell lines and PBMC were incubated at 37°C in a humidified atmosphere with 5% CO_2_, in a HEPES (20 mM)-buffered RPMI 1640 cell culture medium supplemented with 10% fetal bovine serum, 1 mM sodium pyruvate 10 ml/l penicillin/streptomycin (all from Sigma-Aldrich, St. Louis, MO). Cells were incubated in 96-well flat-bottom plates (4×10^4^ or 1.2×10^5^ cells/well for the cell lines or PBMC, respectively) for the viability assessment, 24-well plates (3×10^5^ cells/well for the cell lines) for the flow cytometry analysis or in 100 mm cell culture dishes (2.5×10^7^ or 1×10^8^ cells/well for the cell lines or PBMC, respectively) for the immunoblotting and electron microscopy. Cells were rested for 2 h and then treated with cytarabine in the absence or presence of the autophagy inhibitors bafilomycin A1, chloroquine and 3-methyladenine, or mTOR activator leucine (all from Sigma-Aldrich), as described in Results and Figure legends.

### Cell viability determination

Cell viability was determined by measuring the cellular acid phosphatase activity [25]. At the end of the incubation period (24 or 48 h), culture medium was removed and the acid phosphatase substrate p-nitrophenyl phosphate (10 mM; Sigma-Aldrich) was added. The reaction was stopped after incubation at 37°C for 1 h by addition of 0.1 M NaOH. Color development, corresponding to the number of viable cells, was monitored by automated microplate reader at 405 nm. After subtracting the background value of the cell culture medium, the results were presented relative to untreated control (100% viability).

### Apoptosis analysis

Apoptosis was analyzed by cytometry following double staining with annexin V-FITC and propidium iodide (PI) (BD Pharmingen, San Diego, CA), in which annexin V binds to phosphatidylserine at the surface of apoptotic cells, while PI labels the necrotic cells with membrane damage. Staining was performed according to the manufacturer's instructions and the green (FL1, annexin) and red (FL2, PI) fluorescence was analyzed to evaluate the numbers of viable (annexin^−^/PI^−^), apoptotic (annexin^+^/PI^−^) and necrotic (annexin^+^/PI^+^) cells. DNA fragmentation associated with apoptotic cell death was analyzed by flow cytometry following staining of ethanol-fixed cell with the DNA-binding dye PI, as previously described [26]. The proportion of hypodyploid, apoptotic cells with fragmented DNA (sub-G compartment) was determined after excluding cell aggregates by using a peak fluorescence gate. Caspase activation, as another marker of apoptosis [27], was measured by flow cytometry after labeling the cells with a cell-permeable, FITC-conjugated pan-caspase inhibitor (ApoStat; R&D Systems, Minneapolis, MN) according to the manufacturer's instructions. Caspase activity was assessed by measuring the increase in green fluorescence (FL1). Based on preliminary time-dependence experiments, the flow cytometry analysis of apoptotic changes was performed after 24 h of incubation with cytarabine, using a FACSCalibur flow cytometer and Cell Quest Pro software (BD).

### Measurement of mitochondrial membrane potential and superoxide production

Mitochondrial membrane potential was assessed using DePsipher (R&D Systems), a lipophilic cation that has the property of aggregating upon membrane polarization, forming an orange-red fluorescent compound. If the potential is disturbed, the dye cannot access the transmembrane space and remains or reverts to its green monomeric form. The cells were stained with DePsipher as described by the manufacturer, and the green monomer and red aggregates were detected using a FACSCalibur flow cytometer and Cell Quest Pro software. The results are presented as a green/red fluorescence ratio (FL1/FL2, arbitrarily set to 1 in control samples), the increase of which reflects mitochondrial depolarization. The production of superoxide radical was measured using a superoxide-selective fluorochrome dihydroethidium (DHE) (Life Technologies, Carlsbad, CA). DHE (20 μM) was incubated with the cells for the last 30 min of the treatment and the mean intensity of red fluorescence (FL2), corresponding to superoxide levels, was determined using a FACSCalibur flow cytometer and Cell Quest Pro software. Based on preliminary time-dependence experiments, both mitochondrial membrane potential and superoxide production were analyzed after 24 h of treatment with cytarabine.

### Detection of acidic intracellular vesicles

The acidic vesicles (i.e. lysosomes, autophagolysosomes) were visualized by supravital staining with acridine orange (1 μM; Sigma-Aldrich) for 15 min at 37°C. Cells were analyzed under the inverted fluorescent microscope (Leica Microsystems DMIL, Wetzlar, Germany) using Leica Microsystems DFC320 camera and Leica Application Suite software (version 2.8.1). Depending on their acidity, autophagolysosomes and lysosomes appeared as orange/red fluorescent cytoplasmic vesicles, while nuclei were stained green. Alternatively, acridine orange-stained cells were analyzed on a FACSCalibur flow cytometer using Cell Quest Pro software. Accumulation of acidic vesicles was quantified as red/green fluorescence ratio (mean FL3/FL1). Based on preliminary time-dependence experiments, the analysis was performed after 24 h of treatment with cytarabine.

### Immunoblotting

The cells were lysed in lysis buffer (30 mM Tris-HCl pH 8.0, 150 mM NaCl, 1% NP-40) containing 1 mM phenylmethylsulfonylfluoride and protease/phosphatase inhibitor cocktail (Sigma-Aldrich) on ice for 30 min, centrifuged at 14000 g for 15 min at 4°C, and the supernatants were collected. Equal amounts of protein from each sample were separated by SDS-PAGE and transferred to nitrocellulose membranes (Bio-Rad, Marnes-la-Coquette, France). Following incubation with antibodies against microtubule-associated protein 1 light-chain 3β (LC3β), p62, beclin-1, AMPKα, phospho-AMPKα (Thr172), Akt, phospho-Akt (Ser473), mTOR, phospho-mTOR (Ser2448), p70S6 kinase (p70S6K), phospho-p70S6K (Thr389), ERK, phospho-ERK (Thr202/Tyr204), and actin (all from Cell Signaling Technology, Beverly, MA) as primary antibodies and peroxidase-conjugated goat anti-rabbit IgG (Jackson IP Laboratories, West Grove, PA) as a secondary antibody, specific protein bands were visualized using enhanced chemiluminescence reagent (GE Healthcare, Little Chalfont, UK). The protein levels were quantified by densitometry using ImageJ software and expressed relative to actin (LC3-II, beclin-1, p62) or corresponding total protein signals (phospho-AMPK, phospho-Akt, phospho-mTOR, phospho-p70S6K, phospho-ERK). The results are presented as the fold change in signal intensity compared to that of the untreated control, which was arbitrarily set to 1.

### Transmission electron microscopy

Cells were fixed with 2.5% glutaraldehyde in phosphate-buffered saline, followed by 2% OsO_4_. The thin sections were stained with uranyl acetate and examined using a Morgagni 268D electron microscope (FEI, Hillsboro, OR).

### RNA interference

The transfection of REH cells with small interfering RNA (siRNA) targeting human LC3β, p62, beclin-1 or scrambled control siRNA (all from Qiagen, Valencia, CA), was performed using Lipofectamine 2000 (Life Technologies) according to the manufacturer's instructions. After transfection, cells were allowed to grow 24 h before used for experiments.

### Real-time RT-PCR

Total RNA was extracted with TRIZOL reagent (Life Technologies) and reverse transcribed using MuLV Reverse Transcriptase reverse transcriptase with random hexamer primers (both from Life Technologies) according to the manufacturer's instructions. Real-time RT-PCR was performed in a Realplex^2^ Mastercycler (Eppendorf, Hamburg, Germany) using 96-well reaction plates (Applied Biosystems, Cheshire, UK), TaqMan Universal PCR Master Mix (Applied Biosystems) and TaqMan primers/probes for human Atg4B (Hs00367088_m1), Atg5 (Hs00169468_m1), Atg7 (Hs00197348_m1), Atg12 (Hs00740818_m1), p62 (Hs00177654_m1) and β2-microglobulin (Hs00984230_m1) as a house-keeping gene (all from Applied Biosystems). The amplification conditions were 50°C for 2 min and 95°C for 10 min, followed by 40 cycles of 15 s at 95°C and 1 min at 60°C. All assays were performed in triplicates. Averaged cycle of threshold (Ct) values of β2-microglobulin triplicates were subtracted from Ct values of target genes to obtain ΔCt, and relative gene expression was determined as 2^−ΔCt^. The results were presented relative to the control value, which was arbitrarily set to 1.

### Statistical analysis

The statistical significance of the differences was analyzed by one-way analysis of variance (ANOVA) followed by Student-Newman-Keuls test. A p value less than 0.05 was considered statistically significant.

## Results

### Cytarabine induces apoptosis in REH leukemic cells

**Figure 1:**
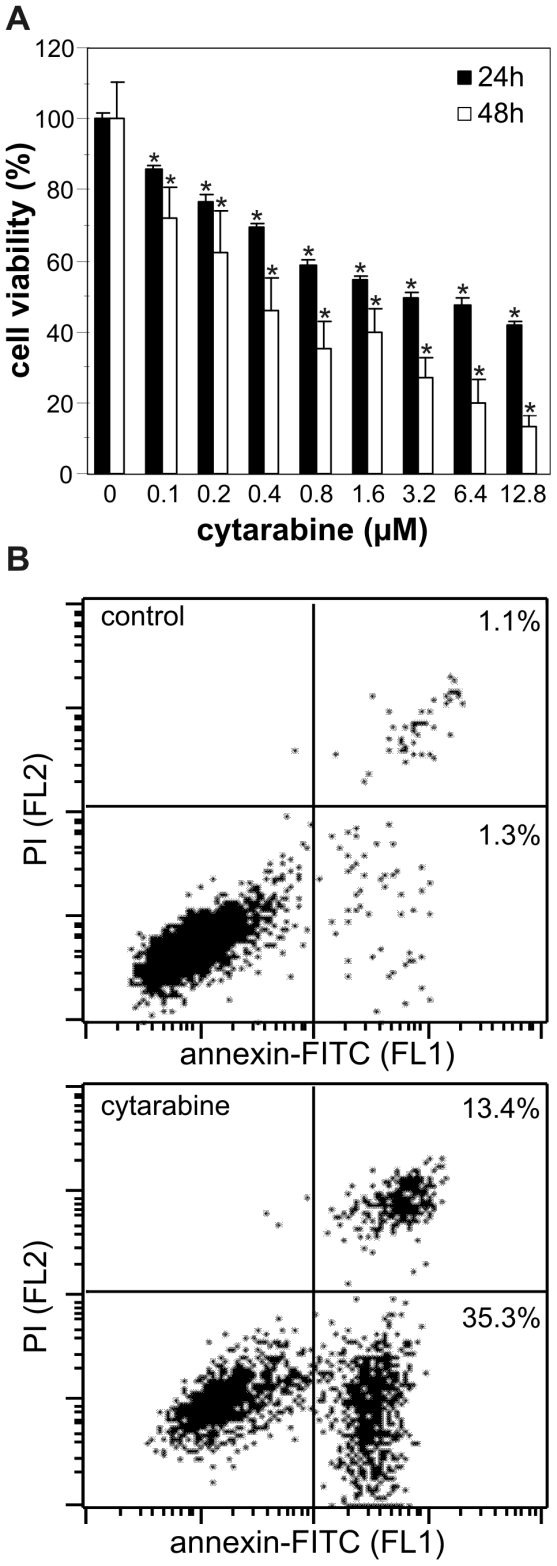
Cytarabine induces apoptosis in REH cells. (A) REH cells were incubated in the presence of different concentrations of cytarabine and cell viability was determined by acid phosphatase assay after 24 h and 48 h. The data are mean ± SD values of triplicate measurements from a representative of three independent experiments (*p<0.05 compared to untreated cells). (B) REH cells were incubated for 24 h with cytarabine (3.2 μM) and phosphatidylserine exposure was determined by flow cytometry after annexin/PI staining. The dot plots from a representative of three independent experiments are presented.

We first investigated the cytotoxicity of cytarabine towards human lymphocytic leukemia REH cell line. The acid phosphatase activity assay revealed that cytarabine reduced the viability of REH cells in a dose-dependent manner, with the IC_50_ concentrations of approximately 3.2 and 0.4 μM after 24 and 48 h, respectively (Fig. 1A). Consequently, 3.2 μM of leukemic drug was used in further experiments. Flow cytometric analysis of cells stained with annexin-FITC and PI demonstrated a significant increase in the proportion of both early apoptotic (annexin^+^/PI^−^) and late apoptotic/necrotic (annexin^+^/PI^+^) cells in cytarabine-treated REH cultures (Fig. 1B). Further analysis of cytarabine-exposed cells confirmed the induction of DNA fragmentation (Fig. S1A) and caspase activation (Fig. S1B), as well as mitochondrial depolarization (Fig. S1C) and oxidative stress (Fig. S1D), which are frequently associated with the initiation of apoptotic cell death [28]. Similar results were obtained with HL-60 (human acute promyelocytic leukemia) and peripheral blood mononuclear cells (PBMC) from CML patients, while PBMC from healthy controls were almost completely insensitive to cytarabine (not shown).

### Cytarabine induces autophagy in leukemic cells

**Figure 2:**
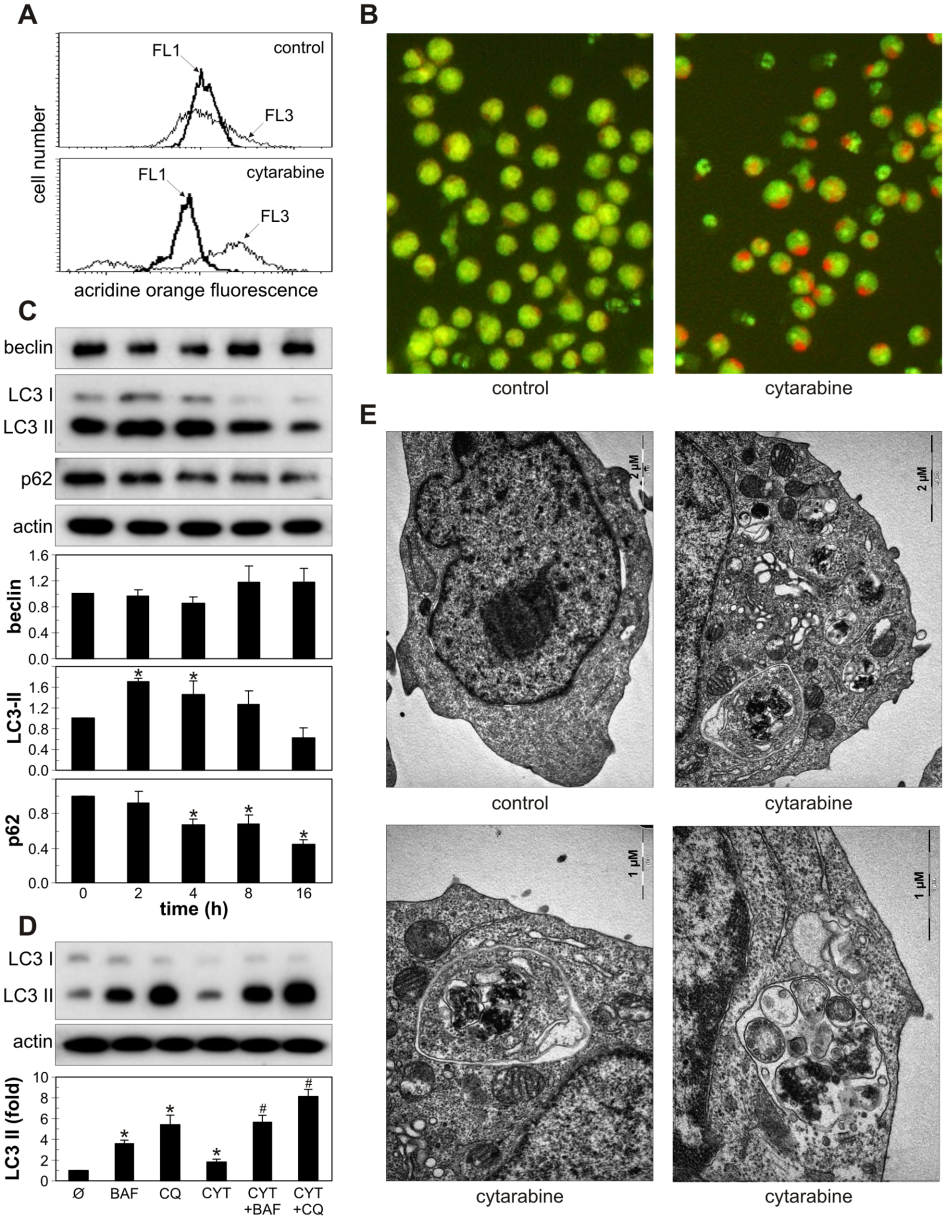
Cytarabine induces autophagy in REH cells. REH cells were incubated with cytarabine alone (3.2 μM) (A-C, E), or with cytarabine in the absence or presence of proteolysis inhibitors bafilomycin A1 (Baf; 10 nM) or chloroquine (Cq; 20 μM) (D). After 16 h, the presence of acridine orange-stained intracellular vesicles was demonstrated by flow cytometry, showing an increase in red/green (FL3/FL1) mean fluorescence ratio (A), or fluorescent microscopy (B). LC3 conversion, beclin-1 and p62 levels were assessed by immunoblotting at the indicated time points (C) or after 8 h (D). The blots from a representative of three independent experiments are presented, while the densitometry data are mean ± SD values (*p<0.05 or ^#^p< 0.05 compared to untreated cells or cells treated with proteolysis inhibitors, respectively). (E) The presence of autophagic vesicles was analyzed after 24 h by electron microscopy and the representative micrographs from two experiments are shown.

**Figure 3:**
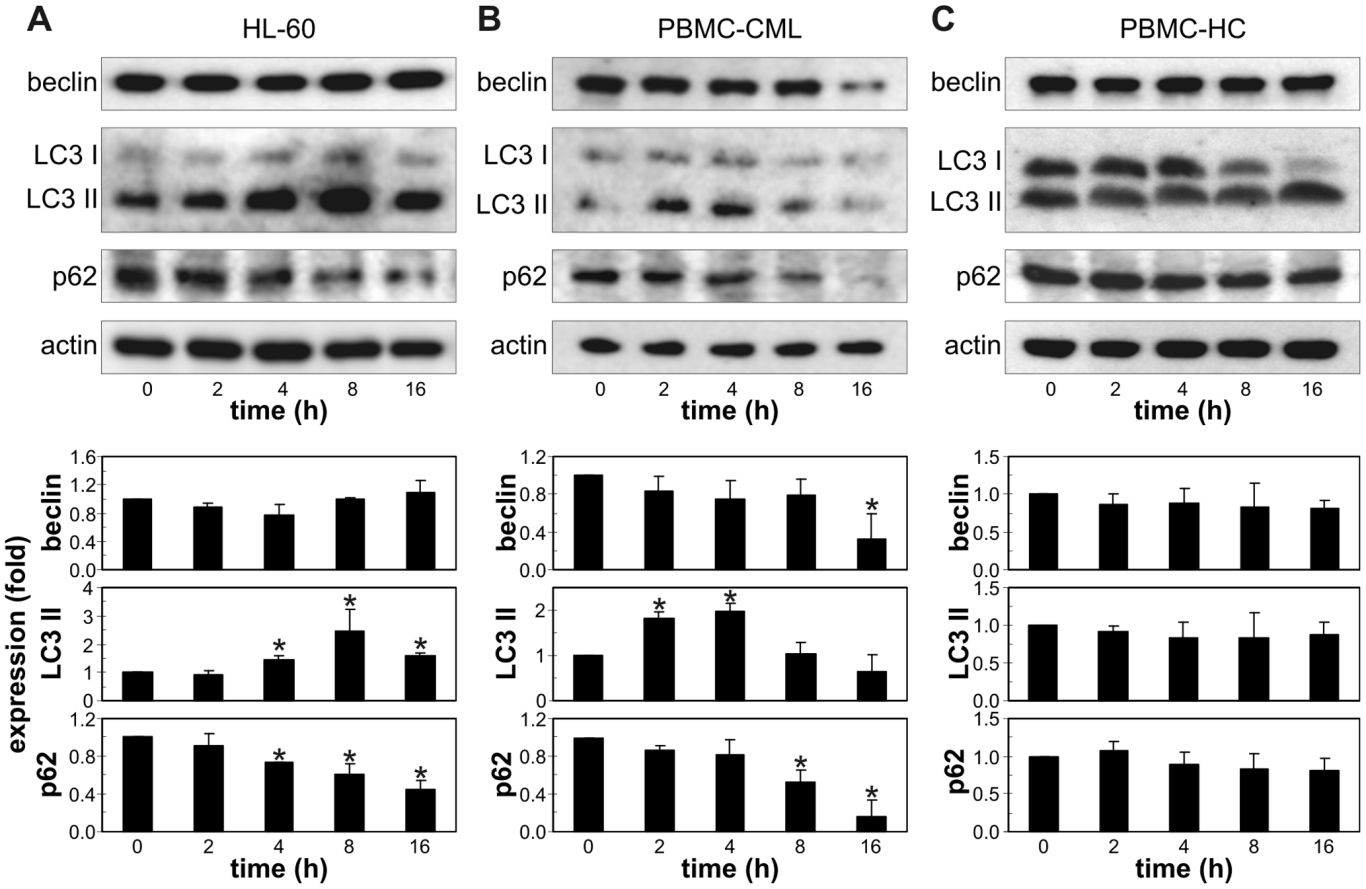
Cytarabine induces autophagy in HL-60 and primary leukemic cells. HL-60 (A), PBMC from CML patients (PBMC-CML) (B) or healthy controls (PBMC-HC) (C) were incubated for the indicated time periods with cytarabine (3.2 μM). The intracellular levels of LC3-II, beclin-1 and p62 levels were assessed by immunoblotting. The representative blots from three independent experiments (A) or three different PBMC samples (B, C) are shown, while the densitometry data are mean ± SD values (*p<0.05 compared to untreated cells).

We next assessed the ability of cytarabine to induce autophagy in leukemic cells. Both flow cytometry and fluorescent microscopy demonstrated an increase in red fluorescence in cytarabine-treated REH cells stained with acridine orange (Fig. 2A, B), indicating the presence of intracellular acidification as one of the hallmarks of autophagic response. This was associated with rapid conversion of LC3-I protein to lipidated, autophagosome-associated LC3-II, which peaked at 2 h and then gradually declined (Fig. 2C), in accordance with its degradation within autophagosomes [4]. Moreover, the treatment with cytarabine strongly decreased the level of p62 (Fig. 2C), a selective target for autophagic degradation [29], confirming the increase in autophagy-mediated proteolysis. On the other hand, the drug failed to increase the expression of the proautophagic protein beclin-1 in REH cells (Fig. 2C). To confirm that the increase in LC3-II levels reflected true induction of autophagy and not the block of LC3-II degradation within autophagolysosomes, we investigated the influence of cytarabine on LC3-II levels in the presence of lysosomal acidification and proteolysis inhibitors bafilomycin A1 and chloroquine. The concentrations of proteolysis inhibitors saturating for LC3-II increase were determined in preliminary experiments (data not shown), while the 8 h-time point was chosen to avoid nonspecific effects of long-term autophagy blockade, as previously recommended [30]. While proteolysis inhibition expectedly increased LC3-II levels in REH cells, treatment with cytarabine additionally increased LC3 conversion (Fig. 2D), suggesting that the drug increased autophagic flux, rather than prevented autophagic proteolysis. In addition, ultrastructural analysis by transmission electron microscopy (Fig. 2E) demonstrated that cytarabine induced an extensive cytoplasmic vacuolization (upper right panel) with many double-membraned autophagosome-like (lower left panel) and single-membraned autophagolysosome-like vesicles (lower right panel) containing cellular material. Cytarabine also stimulated LC3 conversion and p62 degradation in HL-60 and PBMC from CML patients, without increasing beclin-1 expression (Fig. 3A, B). On the other hand, the drug failed to affect the levels of LC3 II, p62 and beclin-1 in PBMC from healthy volunteers (Fig. 3C). These data demonstrate that cytarabine induces autophagic response in different leukemic cells, but not primary leukocytes.

### Cytarabine-induced autophagy in leukemic cells is associated with mTOR inhibition and modulation of Akt, AMPK and ERK

**Figure 4:**
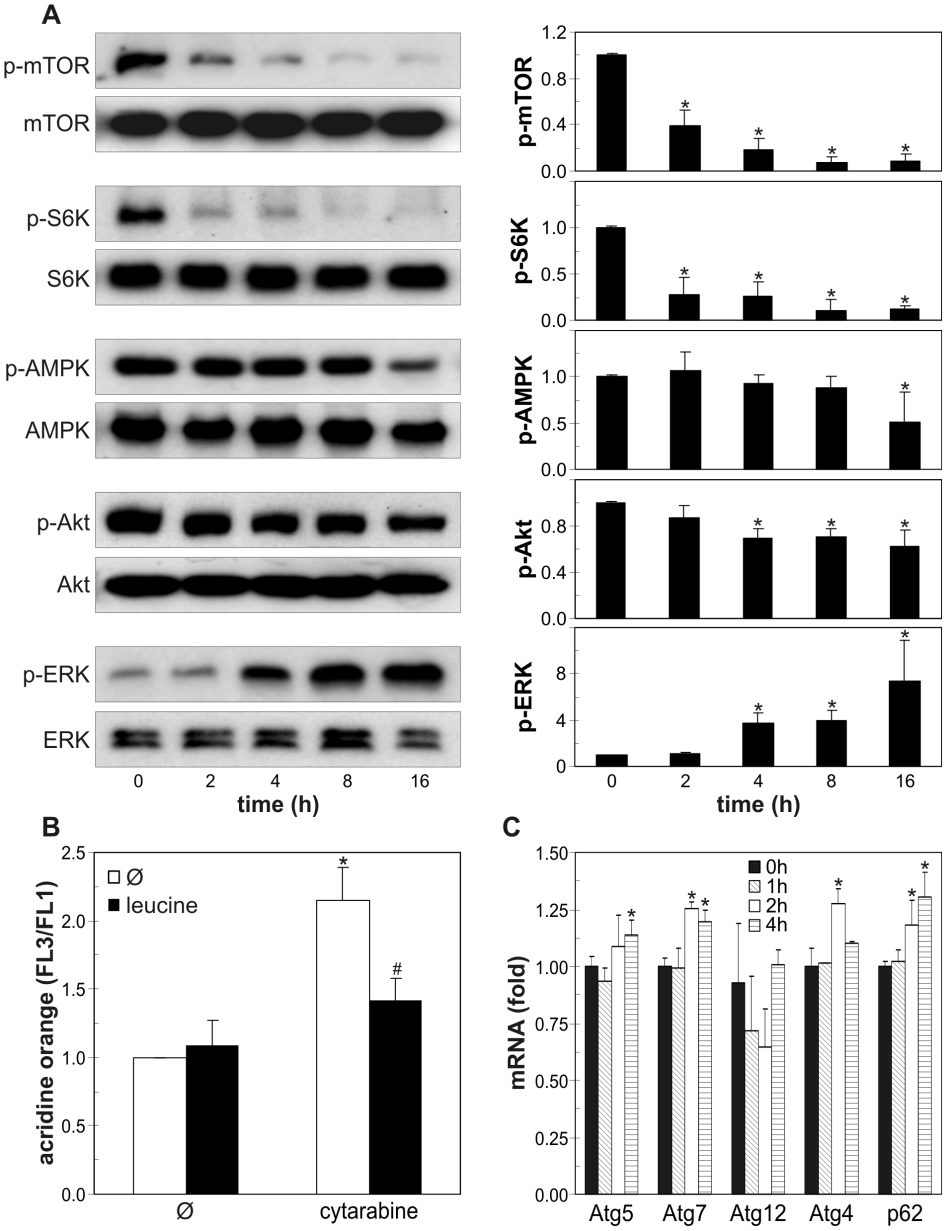
Cytarabine induces Atg expression, inhibits mTOR and modulates Akt/ERK signaling in REH cells. (A, B) REH cells were incubated for the indicated time periods with cytarabine (3.2 μM) and the levels of phosphorylated/total mTOR, p70S6K, AMPK, Akt and ERK were determined by immunoblotting. The representative blots from three independent experiments are shown (A), while the densitometry data are mean ± SD values (*p<0.05 compared to untreated cells) (B). (C, D) REH cells were incubated for 16h (C) or indicated time periods (D) with cytarabine (3.2 μM) in the absence or presence of leucine (2 mM) (C). Intracellular acidification in acridine orange-stained cells was determined by flow cytometry (C), while the amounts of Atg4, Atg5, Atg7, Atg12 and p62 mRNA were analyzed by RT-PCR (D). The data are mean ± SD values from three independent experiments (C) or mean ± SD values of triplicates from a representative of three experiments (D) (*p<0.05 compared to untreated cells).

**Figure 5:**
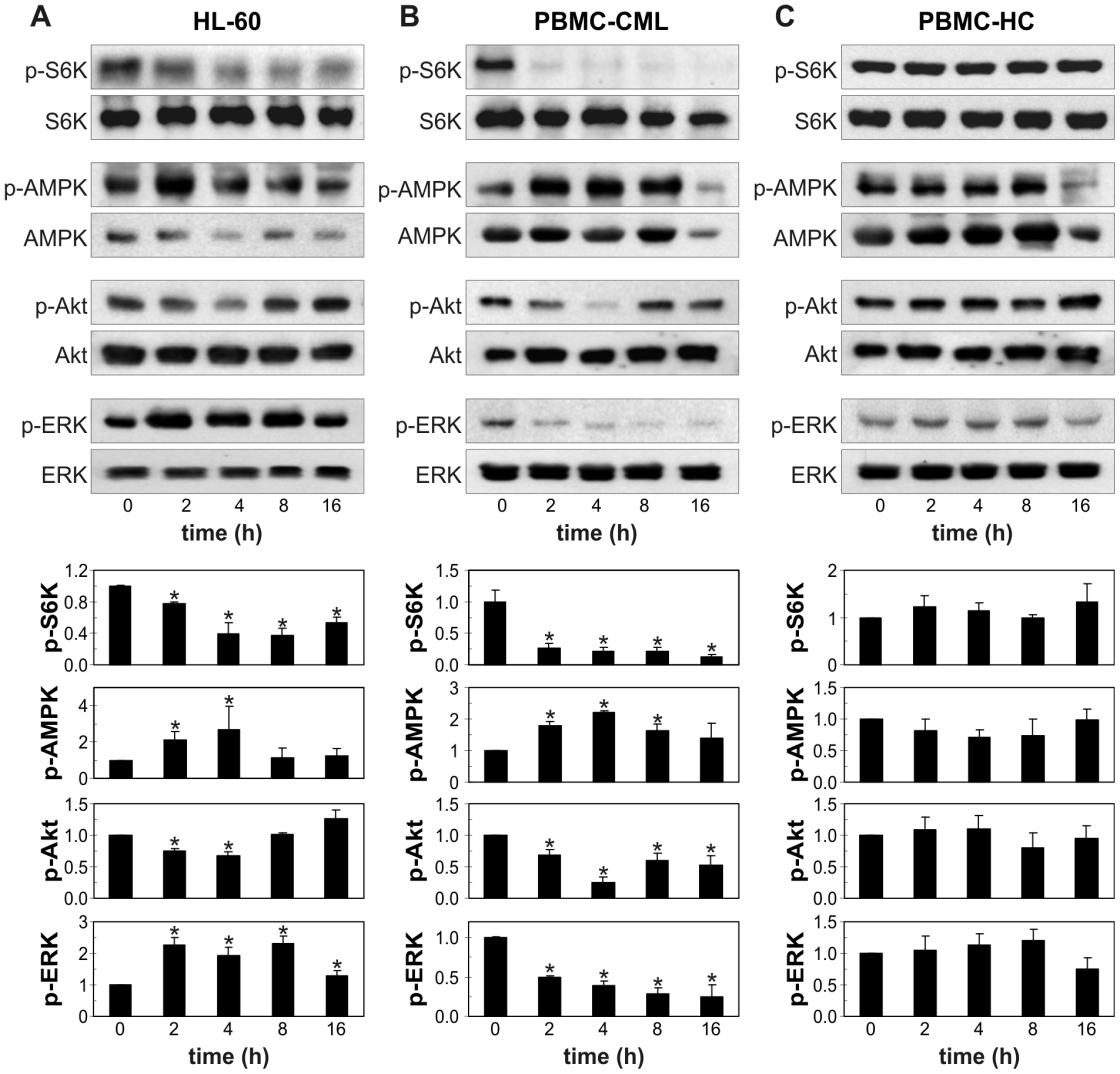
Cytarabine inhibits mTOR and modulates AMPK/Akt/ERK signaling in HL-60 and primary leukemic cells. HL-60 (A), PBMC from CML patients (B) or healthy controls (C) were incubated for the indicated time periods with cytarabine (3.2 μM) and the levels of phosphorylated/total mTOR, p70S6K, AMPK, Akt and ERK were determined by immunoblotting. The representative blots from three independent experiments (A) or three different PBMC samples (B, C) are shown, while the densitometry data are mean ± SD values (*p<0.05 compared to untreated cells).

To further evaluate the mechanisms and signaling pathways involved in cytarabine-triggered autophagy, we assessed the expression of several autophagy-related genes, as well as the activation status of mTOR and its upstream regulators Akt, AMPK and ERK in leukemic cells. The induction of autophagy by cytarabine was associated with the rapid and strong time-dependent reduction in phosphorylation of the autophagy repressor mTOR and its substrate p70S6K (Fig. 4A, B). Leucine, a well known mTOR activator ([31], partly prevented intracellular acidification associated with cytarabine-triggered autophagy in REH cells (Fig. 4C). In addition, RT-PCR analysis demonstrated that cytarabine moderately, but significantly stimulated the expression of Atg4, Atg5, Atg7 and p62, but not Atg12 mRNA, in a time-dependent manner (Fig. 4D). Cytarabine also markedly reduced the activation of p70S6K in HL-60 and primary leukemic cells, but not normal leukocytes (Fig. 5A–F). Antileukemic drug decreased the phosphorylation of mTOR stimulator Akt in all leukemic cells (Fig. 4A, B; 5A–D), but in REH cells this occurred after the reduction of mTOR/p70S6K activation (Fig. 4A, B) and the increase in LC3 conversion (Fig. 2C). On the other hand, the drug strongly stimulated the activity of AMPK, the main intracellular energy sensor with mTOR-inhibiting capacity, in HL-60 and primary leukemic cells (Fig. 5 A–D), but not in REH cells (Fig. 4A, B). Finally, cytarabine stimulated phosphorylation of ERK in HL-60 and REH cells (Fig. 4A, B; 5A, B), but this activation of ERK in REH cells lagged after rapid inhibition of mTOR/p70S6K (Fig. 4A, B). Interestingly, in primary leukemic cells cytarabine strongly decreased activity of ERK (Fig. 5C, D). In agreement with its inability to inhibit p70S6K, the drug failed to change activation status of AMPK, ERK and Akt in primary leukocytes (Fig. 5E, F). These data demonstrate that cytarabine-mediated inhibition of autophagy repressor mTOR in leukemic cells was associated with the cell-specific modulation of mTOR upstream regulators AMPK, Akt and ERK.

### Autophagy inhibition increases cytarabine toxicity to leukemic cells

**Figure 6:**
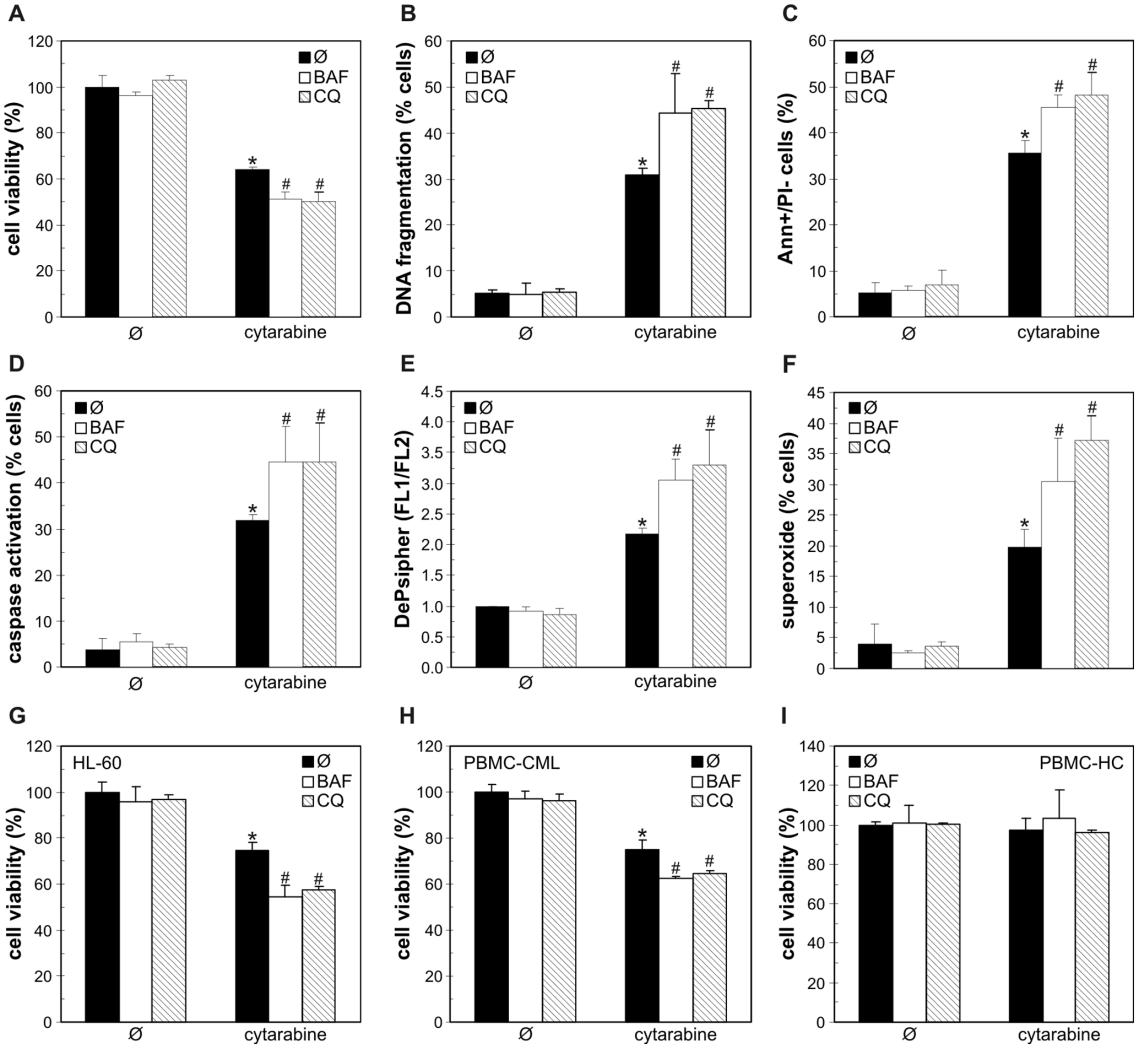
Pharmacological inhibition of autophagy enhances cytarabine-induced apoptosis in leukemic cells. REH (A-F), HL-60 (G) and PBMC from CML patients (H) or healthy controls (I) were incubated for 24 h with cytarabine (3.2 μM) in the presence or absence of the autophagy inhibitors bafilomycin A1 (BAF; 10 nM) or chloroquine (CQ; 20 μM). Cell viability was determined by acid phosphatase test (A, G-I), while DNA fragmentation (B), phosphatidylserine exposure (C), caspase activation (D), mitochondrial depolarization (E) or superoxide production (F) were examined by flow cytometry using appropriate fluorochromes. The data are mean ± SD values of triplicates from a representative of three experiments (A, G-I) or mean ± SD values from three independent experiments (B-F) (*p<0.05 or ^#^p<0.05 compared to untreated cells or cells treated with cytarabine alone, respectively).

**Figure 7:**
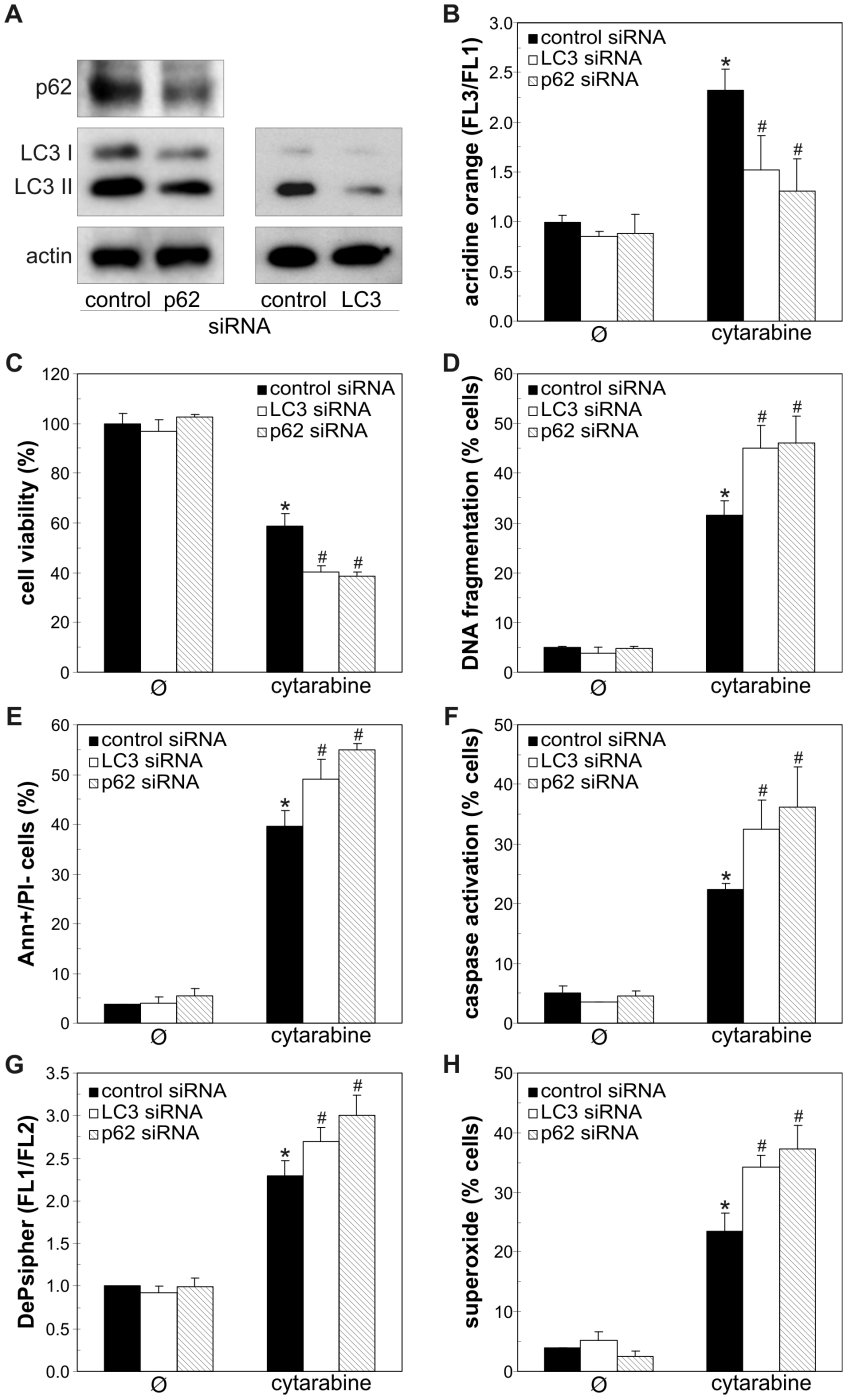
RNA interference-mediated p62 knockdown inhibits autophagy and increases apoptosis in cytarabine-treated REH cells. (A) REH cells were transfected with control, LC3β or p62 siRNA, and p62 and LC3 expression were estimated by immunoblot. (B-H) REH cells transfected with control or p62 siRNA were incubated for 24 h with cytarabine (3.2 µM). Cell viability was analyzed by acid phosphatase assay (C), while the presence of acridine orange (AO)-stained intracellular vesicles (B), DNA fragmentation (D), phosphatidylserine exposure (E), caspase activation (F), mitochondrial depolarization (G) or superoxide production (H) were determined by flow cytometry using appropriate fluorochromes. The data are mean ± SD values of triplicates from a representative of three experiments (C) or mean ± SD values from three independent experiments (B, D-H) (*p < 0.05 or ^#^p < 0.05 compared to untreated or cytarabine-treated control siRNA-transfected cells, respectively).

Finally, to determine the role of autophagy in antileukemic effect of cytarabine, we blocked cytarabine-triggered autophagic response using both pharmacological and genetic approach. Bafilomycin A1 and chloroquine, agents that block autolysosome acidification and autophagic proteolysis [32], markedly increased cytarabine-induced death of REH, HL-60 and primary leukemic cells, but not normal leukocytes (Fig. 6A, G-I). The increase in cytarabine-meditated toxicity towards REH and HL-60 cells was also observed upon co-treatment with 3-methyladenine (Fig. S2), which blocks class III phosphoinositide 3-kinase-dependent formation of autophagosome [33]. The potentiation of cytarabine toxicity by autophagy inhibitors was associated with the increase in DNA fragmentation (Fig. 6B), phosphatidylserine externalization (Fig. 6C), caspase activation (Fig. 6D), mitochondrial depolarization (Fig. 6E) and oxidative stress (Fig. 6F) in REH cells. Accordingly, autophagy knockdown with siRNA against LC3β or autophagic cargo receptor p62 (Fig. 7A), confirmed by a decrease in LC3 protein levels (Fig. 7A) and intracellular acidification (Fig. 7B), further reduced the cell numbers in cytarabine-treated REH cultures (Fig. 7C). The cells transfected with LC3β or p62 siRNA, compared to those transfected with control siRNA, displayed increased DNA fragmentation (Fig. 7D) and phosphatidylserine externalization (Fig. 7E), as well as an increase in caspase activation (Fig. 7F), mitochondrial damage (Fig. 7G) and superoxide production (Fig. 7H) in response to cytarabine. The siRNA-mediated interference with the expression of proautophagic protein beclin-1 also increased the cytotoxicity of cytarabine towards REH cells (Fig. S3). It therefore appears that autophagy induction protected leukemic cells from the coinciding apoptotic cell death.

## Discussion

The present study for the first time demonstrates the ability of cytarabine to trigger mTOR-dependent autophagy in leukemic cells, but not normal leukocytes. Importantly, both pharmacological and genetic inhibition of autophagy increased the sensitivity of leukemic cells to cytarabine-induced apoptotic death, confirming a cytoprotective role of the observed autophagic response.

The occurrence of complete autophagy in cytarabine-exposed leukemic cells was unequivocally demonstrated by assessing multiple autophagy endpoints, including cytoplasmic acidification, morphologic detection of autophagic vesicles, expression of autophagy-related genes, conversion of LC3 with autophagic flux analysis, and digestion of p62 as a selective autophagic target [32]. Interestingly, the levels of the key proautophagic protein beclin-1, required for the initiation of the autophagosome formation [4], were not increased by cytarabine treatment in any of leukemic cells tested. Nevertheless, this basal expression of beclin-1 was apparently required for cytarabine-mediated induction of autophagy, as RNA interference with beclin-1 reduced the autophagic response to cytarabine treatment.

The increase in Atg4, Atg5 and Atg7 expression in cytarabine-treated leukemic cells, while significant, was moderate at best. On the other hand, the downregulation of mTOR/p70S6K activation was almost complete, and autophagy induction was prevented by mTOR activator leucine, indicating that the release from mTOR suppression was the main mechanism for the autophagy induction by cytarabine. These findings are consistent with the view that mTOR regulates autophagy through controlling phosphorylation status, rather than transcription of autophagy-related genes [34], [35]. As for the signals upstream of the observed mTOR inhibition, the activation of AMPK and ERK, as well as inhibition of Akt, have previously been implicated in mTOR inhibition-dependent autophagy in leukemic cells [14], [16], [36]–[39]. Indeed, cytarabine treatment led to a rapid increase in AMPK and ERK activation and decrease in Akt phosphorylation in HL-60 cells, indicating the joint involvement of these signaling pathways in cytarabine-mediated mTOR suppression and subsequent autophagy induction in this leukemic cell line. While AMPK and Akt modulation in cytarabine-treated primary leukemic cells also correlated with mTOR inhibition and autophagy, ERK was downregulated and therefore not responsible for the induction of autophagy by the drug. A third pattern was observed in REH cells in which AMPK was not affected, while the late kinetics of cytarabine-triggered Akt inhibition and ERK activation indicates sustaining, rather than causative role in mTOR suppression and autophagy induction. Another plausible candidate for mediating cytarabine-triggered autophagy is tumor suppressor p53, which has been implicated in the induction of mTOR-dependent autophagic response in stressed cells [40], and is upregulated in leukemic cells by cytarabine treatment [41]. Therefore, cytarabine-induced changes in signaling molecules converging to mTOR inhibition and subsequent induction of autophagy seem to be leukemic cell type-specific and require further investigation.

Similarly to previously reported results [42], [43], cytarabine-induced oxidative stress and mitochondrial depolarization in the present study were associated with several apoptotic markers, including caspase activation, phosphatidylserine exposure and DNA fragmentation. However, the causal role of oxidative stress/mitochondrial depolarization in cytarabine-triggered apoptosis remains to be directly confirmed, as these events may merely be reflecting the effects, rather than cause of cell death. Nevertheless, it is conceivable to assume that oxidative stress was responsible for autophagy induction by cytarabine, as production of ROS, particularly superoxide, has been implicated in the activation of autophagic response [44]–[46]. To assess the role of cytarabine-triggered autophagic response in the concomitant induction of apoptotic death, we employed both pharmacological and genetic donwregulation of autophagy. In accordance with previous findings in leukemic cell lines [7], [10], [47], [48], the RNA interference with beclin-1 and LC3β, which are required for autophagosome formation, or p62, the cargo receptor delivering ubiquitinated proteins to autophagic compartments, efficiently impaired autophagy induction in REH cells. The inhibition of early autophagy events with beclin-1/LC3β/p62 siRNA or 3-methyladenine, as well as the interference with autophagic digestion with proteolysis inhibitors, significantly increased cytarabine-induced cell death, thus indicating a protective role of cytarabine-induced autophagy in leukemic cell lines (REH, HL-60) and primary leukemic cells. The inhibition of basal autophagy, on the other hand, was not overtly detrimental to leukemic cells, probably due to a relatively short incubation period. Similarly to our results, interference with the autophagic response increased the cytotoxicity of daunorubicin, flavopiridol, triciribine and tyrosine kinase inhibitors towards leukemic cells [11]–[14]. On the other hand, autophagy was apparently involved in antileukemic effects of arsenic trioxide, dexamethasone, decitabine and acadesine [6]–[10], indicating that the role of autophagy in survival/death of leukemic cells depends on the cytotoxic agent.

While for the first time demonstrating the cytoprotective autophagy in leukemic cells exposed to cytarabine alone, our data are consistent with previous findings that autophagy induced by high-mobility group protein B1 and imatinib mesylate can reduce the in vitro antileukemic action of the drug [22], [23]. As for the mechanisms responsible for the cytoprotective effect of autophagy in the present study, it has been proposed that autophagic response may act as a negative feedback mechanisms for removal of oxidized proteins and dysfunctional ROS-producing mitochondria, thus preventing their involvement in cellular injury [49]. Indeed, the inhibition of autophagy in cytarabine-treated leukemic cells markedly increased superoxide production and subsequent mitochondrial damage, leading to caspase activation and apoptotic DNA fragmentation. While this indicates the involvement of oxidative stress attenuation in autophagy-mediated protection from cytarabine, the precise mechanisms of autophagy-apoptosis interplay in the cytarabine cytotoxicity remain to be elucidated.

In conclusion, we demonstrate that inhibition of autophagy increases apoptotic death of leukemic cells treated with cytarabine at the dose (3.2 μM) achievable in the blood of leukemic patients [50]. While the similar response was not observed in healthy blood leukocytes, it should be noted that normal bone marrow cells, which are difficult to obtain due to ethical issues, would be more appropriate controls. Therefore, it remains to be assessed if protective autophagy is induced similarly in normal blood progenitor cells treated with cytarabine, since the use of autophagy inhibitors could then be counter-productive. Chloroquine, a clinically approved autophagy inhibitor that blocks lysosomal proteolysis [51] is currently in phase II clinical trials for treatment of leukemia and multiple myeloma in combination with cyclophosphamide, bortezomib or imanitib (Trial #NCT01438177, ISCRTN No. 61568166), and the in vitro doses of chloroquine used in the present study are within one order of magnitude of the peak blood concentrations achievable in humans [53]. Therefore, our results indicate that a similar approach might be used to sensitize leukemic cells to cytarabine, thus decreasing its effective dose and ameliorating side effects. Moreover, in light of the possible use of mTOR inhibitors as adjuvant therapy in leukemia [52], our data suggest that their efficiency in combination with cytarabine might be further improved by inhibition of the concomitant autophagic response. However, caution is clearly warranted due to opposing, context-dependent actions of autophagy in regulating leukemic cell death and survival.

## Supporting Information

Figure S1 **Cytarabine induces apoptosis-related changes in REH cells.** (A-D) REH cells were incubated for 24 h with cytarabine (3.2 μM) and DNA fragmentation (A), caspase activation (B), mitochondrial depolarization (C) or superoxide production (D) were determined by flow cytometry using appropriate fluorochromes. The histograms from a representative of three independent experiments are presented.
(TIF)

Figure S2 **3-methyladenine increases the cytotoxicity of cytarabine towards leukemic cell lines.** REH (A) or HL-60 (B) cells were incubated for 24 h with cytarabine (3.2 μM) in the presence or absence of the autophagy inhibitor 3-methyladenine (5 mM). Cell viability was determined by acid phosphatase test and the data are presented as mean ± SD values of triplicates from a representative of three experiments (*p<0.05 or ^#^p<0.05 compared to untreated cells or cells treated with cytarabine alone, respectively).
(TIF)

Figure S3 **RNA interference with beclin-1 increases the cytotoxic action of cytarabine in REH cells.** (A) REH cells were transfected with control or beclin-1 siRNA and the decrease in beclin-1 expression was confirmed by immunoblot. (B) REH cells transfected with control or beclin-1 were incubated for 24 h with different concentrations of cytarabine and cell viability was analyzed by acid phosphatase assay. The data are mean ± SD values of triplicates from a representative of three experiments (*p<0.05 or^ #^p<0.05 compared to untreated or cytarabine-treated control siRNA-transfected cells, respectively).
(TIF)
