# Supplementary figures and images for: Inhibition of mTOR-Dependent Autophagy Sensitizes Leukemic Cells to Cytarabine-Induced Apoptotic Death
Source: PLoS One. 2014 Apr 8;9(4):e94374. doi: 10.1371/journal.pone.0094374 (PMC3979773; doi:10.1371/journal.pone.0094374)

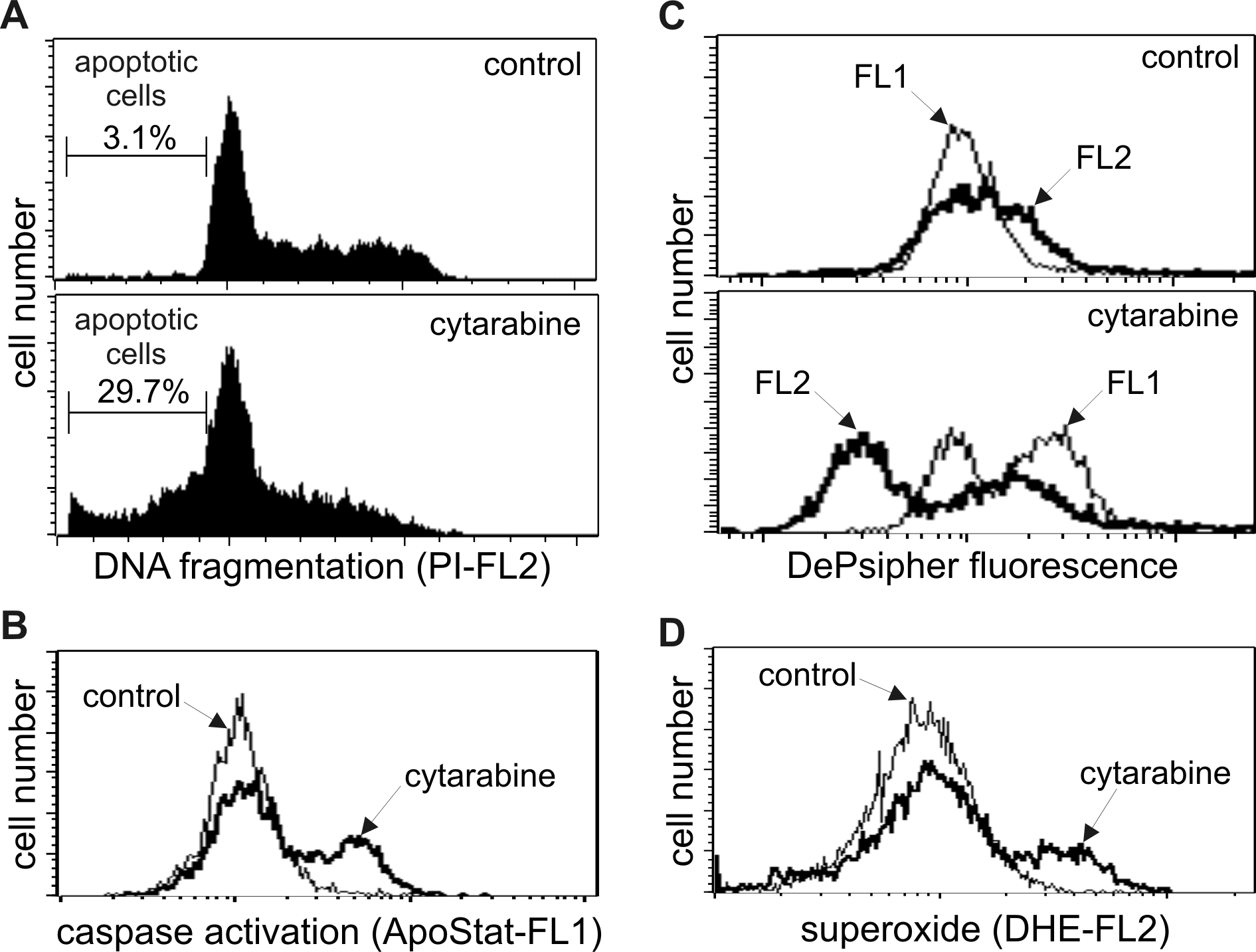

Supplement: Figure S1 — Cytarabine induces apoptosis-related changes in REH cells. (A-D) REH cells were incubated for 24 h with cytarabine (3.2 μM) and DNA fragmentation (A), caspase activation (B), mitochondrial depolarization (C) or superoxide production (D) were determined by flow cytometry using appropriate fluorochromes. The histograms from a representative of three independent experiments are presented. (TIF) [file pone.0094374.s001.tif]

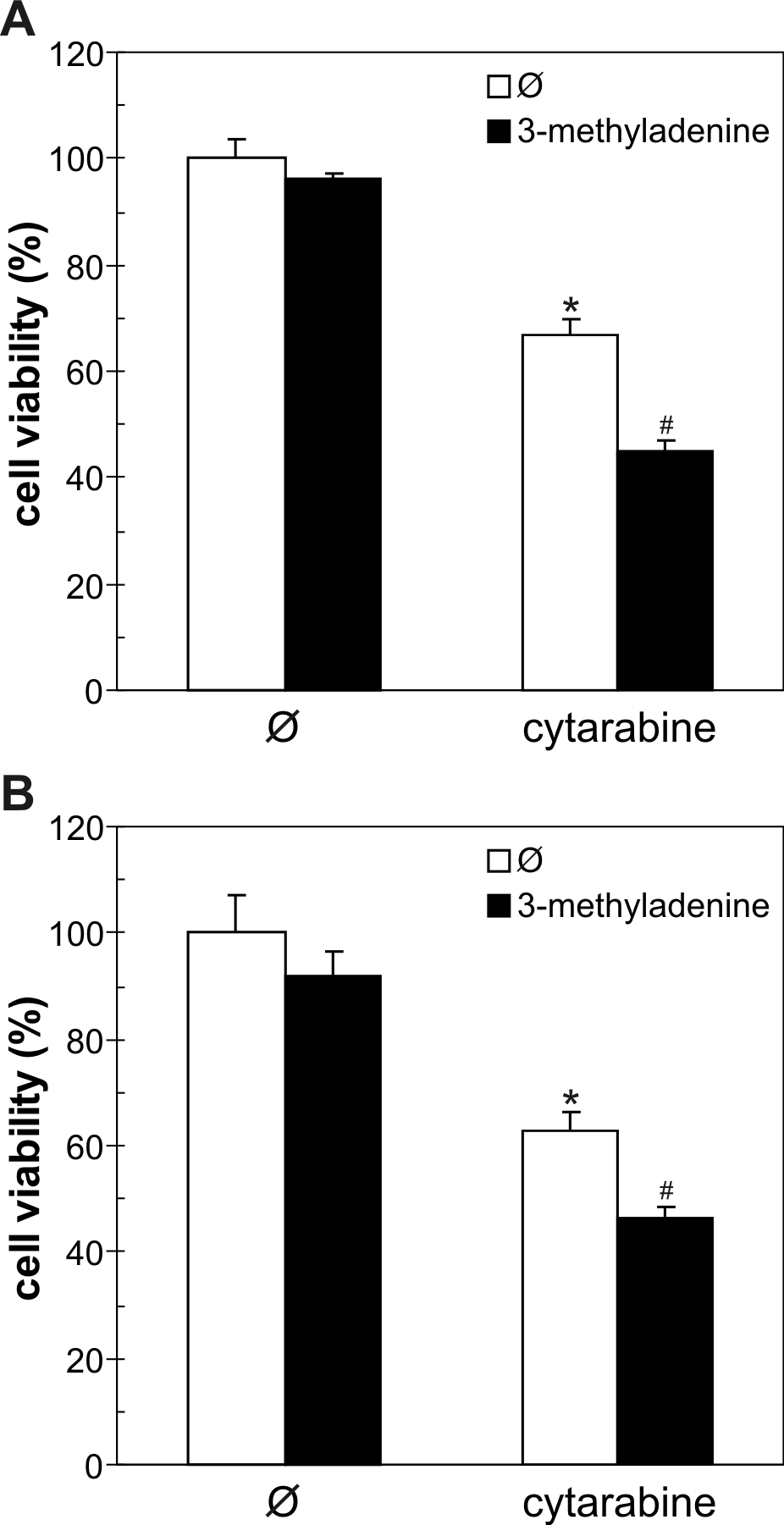

Supplement: Figure S2 — 3-methyladenine increases the cytotoxicity of cytarabine towards leukemic cell lines. REH (A) or HL-60 (B) cells were incubated for 24 h with cytarabine (3.2 μM) in the presence or absence of the autophagy inhibitor 3-methyladenine (5 mM). Cell viability was determined by acid phosphatase test and the data are presented as mean ± SD values of triplicates from a representative of three experiments (*p<0.05 or #p<0.05 compared to untreated cells or cells treated with cytarabine alone, respectively). (TIF) [file pone.0094374.s002.tif]

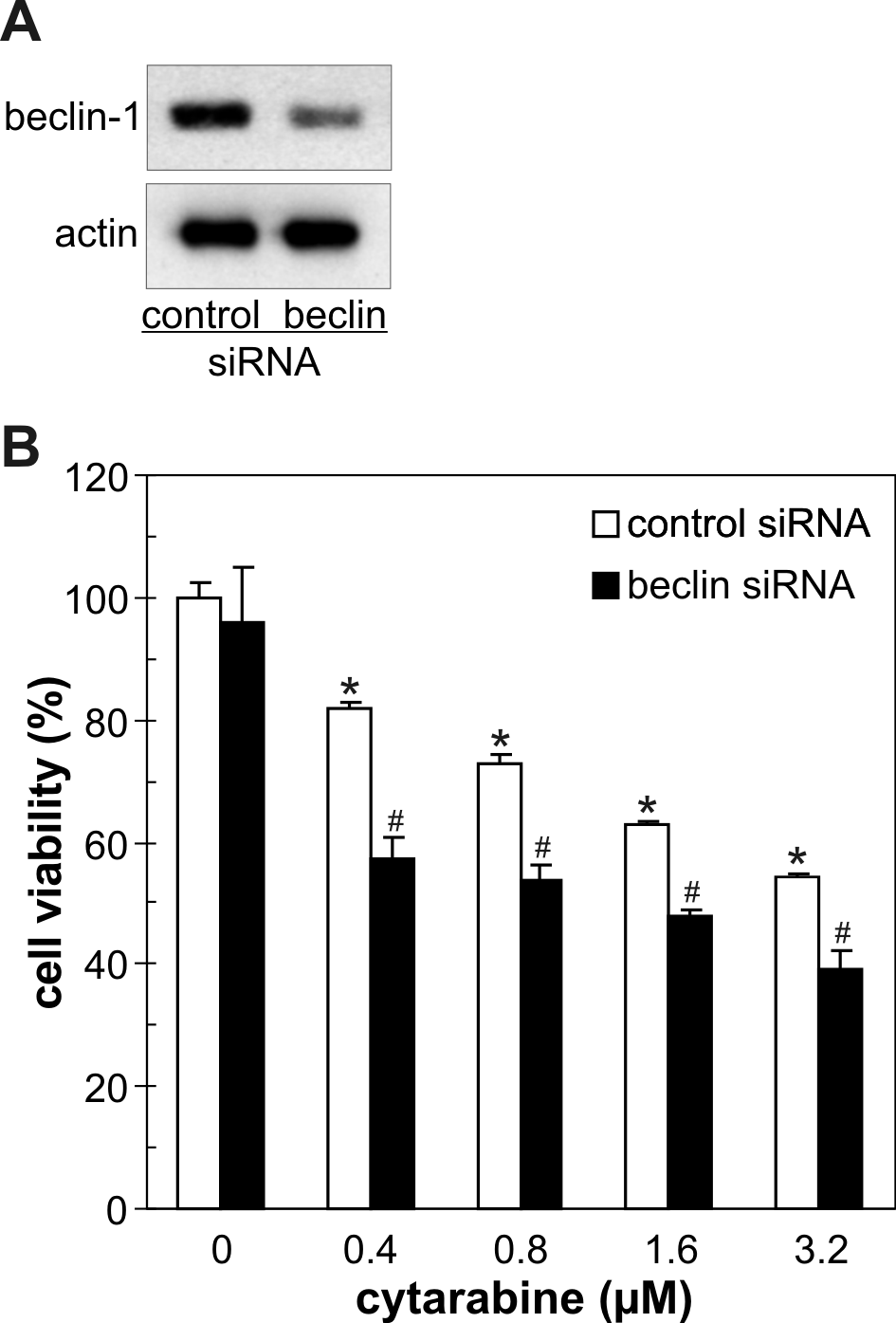

Supplement: Figure S3 — RNA interference with beclin-1 increases the cytotoxic action of cytarabine in REH cells. (A) REH cells were transfected with control or beclin-1 siRNA and the decrease in beclin-1 expression was confirmed by immunoblot. (B) REH cells transfected with control or beclin-1 were incubated for 24 h with different concentrations of cytarabine and cell viability was analyzed by acid phosphatase assay. The data are mean ± SD values of triplicates from a representative of three experiments (*p<0.05 or #p<0.05 compared to untreated or cytarabine-treated control siRNA-transfected cells, respectively). (TIF) [file pone.0094374.s003.tif]
